# *Aryl hydrocarbon receptor* (*Ahr*)‐dependent *Il‐22* expression by type 3 innate lymphoid cells control of acute joint inflammation

**DOI:** 10.1111/jcmm.16433

**Published:** 2021-03-18

**Authors:** Ramzi Nehmar, Louis Fauconnier, Jose Alves‐Filho, Dieudonnée Togbe, Aurore DeCauwer, Seiamak Bahram, Marc Le Bert, Bernhard Ryffel, Philippe Georgel

**Affiliations:** ^1^ Laboratoire d’ImmunoRhumatologie Moléculaire Institut national de la santé et de la recherche médicale (INSERM) UMR_S 1109 Institut thématique interdisciplinaire (ITI) de Médecine de Précision de Strasbourg Transplantex NG Faculté de Médecine Fédération Hospitalo‐Universitaire OMICARE Fédération de Médecine Translationnelle de Strasbourg (FMTS) Université de Strasbourg Strasbourg France; ^2^ ArtImmune SAS Orleans France; ^3^ Department of Pharmacology Ribeirao Preto Medical School, University of Sao Paulo Ribeirao Preto Brazil; ^4^ Laboratory of Experimental and Molecular Immunology and Neurogenetics (INEM) UMR 7355 CNRS‐University of Orléans Orleans France

**Keywords:** acute arthritis, aryl hydrocarbon receptor, IL‐22, inflammation

## Abstract

The aryl hydrocarbon receptor (AHR) controls several inflammatory and metabolic pathways involved in various diseases, including the development of arthritis. Here, we investigated the role of AHR activation in IL‐22‐dependent acute arthritis using the K/BxN serum transfer model. We observed an overall reduction of cytokine expression in *Ahr*‐deficient mice, along with decreased signs of joint inflammation. Conversely, we report worsened arthritis symptoms in *Il‐22* deficient mice. Pharmacological stimulation of AHR with the agonist VAG539, as well as injection of recombinant IL‐22, given prior arthritogenic triggering, attenuated inflammation and reduced joint destruction. The protective effect of VAG539 was abrogated in *Il‐22* deficient mice. Finally, conditional *Ahr* depletion of *Rorc*‐expressing cells was sufficient to attenuate arthritis, thereby uncovering a previously unsuspected role of AHR in type 3 innate lymphoid cells during acute arthritis.

## 1 INTRODUCTION

Recent progresses in genome analyses technologies enabled the identification of more than 300 genes involved in autoimmune diseases.^1^ However, for a large proportion of patients, causal variants in these genes are not observed, leading to the ‘missing heritability’ concept.^2^ Epigenetic modifications, environmental factors or individual's microbiota are frequently considered as major, non‐genetic, additional drivers of autoimmunity. Indeed, the prevalence of autoimmune diseases is increasing worldwide, a feature that has been linked to dramatic changes in human behaviour such as food consumption, but also to worsening air pollution.^3^

Environmental pollutants are suspected to trigger several autoimmune diseases, including rheumatoid arthritis (RA).^4^ Notably, polycyclic aromatic hydrocarbons (PAH), dioxin (TCDD), phthalates or alkylphenols activate the transcription factor aryl hydrocarbon receptor (AHR),^5^, ^6^, ^7^ which has been involved in RA pathophysiology in mice.^8^, ^9^ Interestingly, AHR also senses tryptophan metabolites including those of bacterial origin.^10^ This suggests that AHR can participate in the surveillance and homeostasis of the microbiota, the composition of which may also impact on RA pathogenesis.^11^ Ligand‐activated AHR translocates into the nucleus forming a transcriptional complex leading to ligand‐dependent transcription of several genes regulating immune responses, such as *Rort*, *Il‐17* or *FoxP3*.^6^, ^12^, ^13^, ^14^, ^15^

In the intestine, AHR regulates the innate immune response through the modulation of innate lymphoid cells (ILC3) secreting IL‐22 following *Il‐22* gene transcriptional induction.^16^ In mice, IL‐22 is part of the RORt‐dependent Th17 cell differentiation programme.^17^ IL‐17 and IL‐22 are known promoters of allergic diseases, as demonstrated by their role in allergic lung inflammation^18^, ^19^, ^20^ which is dependent on AHR activation.^21^ However, while a role of IL‐22 and IL‐17 *via* PTEN and miR‐155 has been demonstrated in RA mouse models,^22^, ^23^ the precise role of the AHR‐IL‐22 axis in RA is still debated, particularly in an acute model of joint inflammation. Here, we used the K/BxN serum transfer arthritis model (STA) to explore the impact of *Ahr*‐dependent *Il‐22* expression on acute joint inflammation. In this model, preformed antibodies directed towards Glucose‐6‐phosphate isomerase induces systemic inflammatory symptoms, including polyarthritis, dependent on IL‐1β and TNFα.^24^, ^25^, ^26^ We showed that *Ahr* transcription is activated in the synovial tissue upon serum transfer. The resulting arthritis exhibits reduced symptoms in *Ahr*‐deficient mice expressing lower IL‐22 levels. Furthermore, administration of both the AHR agonist VAG539^27^ enhancing IL‐22, and rIL‐22 attenuated serum transfer arthritis. Of note, VAG539 effects were IL‐22 dependent. Finally, using various genetically modified mouse models, we provide evidence that (i) IL‐22 has a protective function in this model and (ii) that type 3 innate lymphoid cells (ILC3) expressing *Ahr* are important regulators of acute joint inflammation.

## 2 MATERIALS AND METHODS

### 2.1 Mice and reagents

*Ahr* ^−/−^ were provided by Frank J. Gonzalez (Center for Cancer Research, National Cancer Institute) and floxed *Ahr* were obtained from Christopher A Bradfield (University of Wisconsin‐Madison, WI). *Il‐22* ^−/−^,^28^ *Rorc* KO, Rorc‐Cre and Rorc‐GFP reporter mice from Gerard Eberl (Institute Pasteur) and ILC3 specific Ahr‐Rorc mice were generated by crossing floxed *Ahr* mice with Rorc‐Cre mice (*Ahr^f/f^xRorc‐Cre*). Colonies were maintained on a C57Bl/6 genetic background. Mice were bred and housed in our specific pathogen free animal facility at Transgenose Institute (TAMM‐CNRS, UPS 44 under the agreement D‐45‐234‐6, 2014). 5‐6 female mice, 7 to 10 weeks old were used in all the experiments. They were maintained in a temperature controlled (23°C) facility with a strict 12h light/dark cycle and were given free access to food and water. Animal experiments were performed according to the French Institutional Ethic Committee under the agreement CLE CCO 2015‐1088.

Anti‐IL‐22 antibody (AM22.1, mouse anti‐mouse, 14 µg/mouse) was a gift from Laure Dumoutier (Université Catholique de Louvain, Belgium). Antibodies were injected intraperitoneally 1h before and at day 3 after administration of K/BxN serum. RhIL‐22 was from Generon Ltd Shanghai (Dr Xiaoqiang Yan). The AHR agonist, VAG539, was given by oral gavage daily at 50mg/kg (as advised by Dr José Carballido, Novartis, Basel, Switzerland).^27^

### 2.2 K/BxN serum arthritis induction

Two hundred microliter of serum harvested from K/BxN mice were injected by the intravenous route on day 0 and day 1. Joint inflammation is visible at day 3 and peaks at day 6.^29^ Ankle thickness of the fore and hind limbs was measured daily with a caliper and the clinical score (0 = no swelling or erythema, 1 = slight swelling and/or erythema, 2 = low‐to‐moderate oedema, 3 = pronounced oedema with limited use of the joint, and 4 = excessive oedema with joint rigidity) reflects daily observations of the extent of swelling and reddening of the joints. The clinical indices for all four paws were added as a composite score.

### 2.3 Cytokine quantification

Six days after K/BxN serum injection, synovial tissues of the two posterior ankles were dissected and homogenized in 500µl of buffer‐containing protease inhibitors. IL‐6, IL‐10, IL‐22, keratinocyte‐derived chemokine (KC/CXCL1) and myeloperoxydase concentrations were measured by enzyme‐linked immunosorbent assay (ELISA) using commercial kits (DuoSet, R&D Systems) as previously described.^19^

### 2.4 Microscopy

Mice were euthanized at day 10. Ankle joints were removed and fixed for 72 hours with 10% paraformaldehyde (pH 7.2). The ankle joint was then incubated in 10% EDTA at pH 7.2 for 10 days at room temperature to decalcify the bone. The samples were rinsed in PBS and dehydrated; embedded in paraffin, cut at 3 µm and stained with haematoxylin and eosin (H&E). To eliminate potential bias, the slides were scored by independent observers. The slides were graded using various parameters, such as severity of synovial hyperplasia (pannus formation), cellular exudates, and cartilage depletion/bone erosion, each scored 0 to 5, and extent of synovial infiltrate, scored 0‐5, with higher scores indicating greater infiltration. The grades for all parameters were subsequently summed to obtain an arthritis index, with results expressed as the median arthritis score.

### 2.5 Gene expression analyses

Synovial tissue was collected at 24h, snap‐frozen in liquid nitrogen and kept at −80°C. Total RNA was isolated and homogenized with 1 mL of TRI Reagent^®^ (Sigma) using TRIzol/Chloroform extraction. RNA was then precipitated in isopropanol, washed with 75% ethanol and resuspended in RNase‐free water. Reverse transcription was performed on 1 µg of RNA using GoScript Reverse transcription system (Promega). Quantitative real‐time PCR were realized on cDNA obtained using primers for *Ahr, Cyp1a1* and *Il‐22* (Qiagen), GoTaq^®^ qPCR‐Master Mix (Promega) and detected on a Stratagene Mx3005P (Agilent technologies). At the end of the PCR amplification, a DNA melting curve analysis was carried out to confirm the presence of a single amplicon. *Gapdh* expression was used for normalization of transcript levels. Relative mRNA levels were determined using (2^−ΔΔCt^) method, determined by comparing () the PCR cycle thresholds (Ct) for the gene of interest and *Gapdh* (ΔCt) and () ΔCt values for treated and control groups (ΔΔCt).

### 2.6 Flow cytometry

Synovial tissue was collected at day 4 and digested in RPMI 1640 medium containing U/mL penicillin, U/mL streptomycin, 1 mg/mL DNase I (Sigma) and 125 μg liberase (Roche) for 1 h at 37°C under rotation. After digestion, RPMI 1640 supplemented with 10% FCS was added. Cells were dissociated by passage through a 70 μm cell strainer and centrifuged at 400× *g* for 5 min at 4°C. Pellet was resuspended in red blood cell lysis buffer (Stem cell Technologies) and incubated for 10 min on ice. Lysis was stopped by addition of RPMI 1640 and centrifuged again at 400*g* for 5 min at 4°C. Pellet was resuspended in RPMI 1640 supplemented with 10% FCS and passed through a 40 μm cell strainer. ILC3 were identified using a cocktail of antibodies and were defined as CD45+, CD127+, ICOS+and RoRγt+. Rorc‐GFP expressing cells were analysed by flow cytometry (FACS Canto 2, BD).

### 2.7 Statistical analyses

Data were analysed using Prism version 5 (Graphpad Software). The non‐parametric Kruskal‐Wallis test with Dunn's multiple comparison test or the parametric one‐way ANOVA test with multiple Bonferroni's comparison test were used. Mann‐Whitney *U* test was used to compare two groups of values. Values are expressed as mean ± SEM. Statistical significance was defined at a *P*‐value < 0.05.

## 3 RESULTS

### 3.1 Attenuated arthritis in the K/BxN serum transfer model in *Ahr*‐deficient mice

**FIGURE 1:**
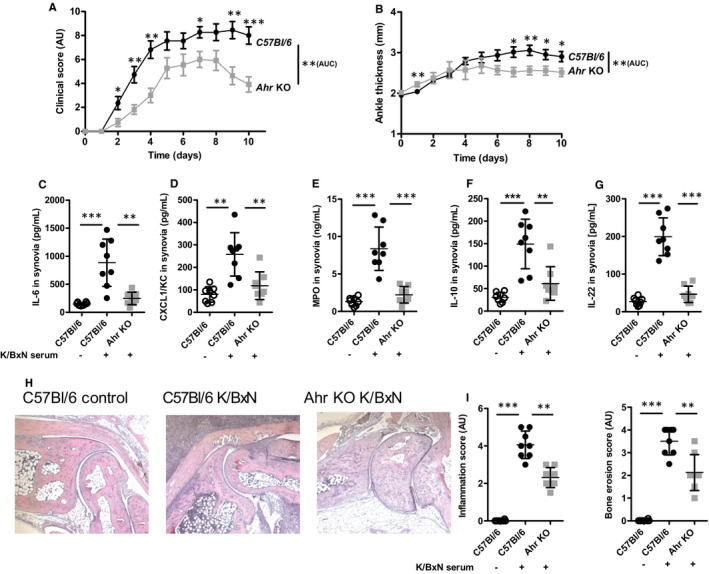
Attenuated K/BxN serum arthritis in the absence of *Ahr*. A, Clinical score (expressed in arbitrary units, AU) and B, ankle swelling (in mm). Cytokine expression in the synovium C, IL‐6 (in pg/mL), D, CXCL1 (in pg/mL), E, MPO (in ng/mL), F, IL‐10 (in pg/mL) and G, IL‐22 (in pg/mL). H, Representative histological pictures of the joint in control mice and following K/BxN serum transfer in wild type (C57Bl/6) and *Ahr* KO animals at day 6. I. Inflammation and bone erosion scores (in arbitrary units, AU) quantified from histological analysis at day 10. Mean values +/− standard deviation are shown (n = 8 to 10 mice/group). Representative data from two independent experiments are shown. Data were analysed following a Mann‐Whitney *U* test. *P <.05**P <.01***P <.001. AUC: area under the curve

The role of the aryl hydrocarbon receptor (AHR) in autoimmunity^30^, ^31^ and in RA in particular^32^ has previously been explored in the collagen‐induced arthritis model, which highlighted the role of T cells^8^ and IL‐17^9^ in this process. However, the precise mechanisms linking AHR signalling to inflammatory responses remains poorly described. Here, we used the acute and transient model provided by the transfer of arthritogenic serum harvested from K/BxN donor mice into C57/Bl6 controls and *Ahr*‐deficient recipients^24^ to better understand the molecular and cellular players in the AHR‐mediated control of inflammation. As seen in Figure 1A,B, *Ahr*‐deficient mice display reduced arthritis symptoms, as demonstrated by a diminished clinical score and ankle thickness upon serum transfer, in agreement with other models like collagen‐induced arthritis or antigen‐induced arthritis.^8^, ^9^ Interestingly, the expression of inflammatory cytokines also appeared affected by loss of *Ahr*. This is the case of IL‐6 (Figure 1C) which does not participate in disease progression in this model, but which quantification represents a reliable marker of inflammation.^25^ Furthermore, reduced IL‐6 concentration in the synovia is in line with AHR‐dependent activation of the NF‐B signalling pathways and control of *Il‐6* gene expression.^33^ In addition, the expression and secretion of chemokines such as CXCL1 is also diminished, which is in agreement with reduced expression of myeloperoxydase (MPO), an important marker of neutrophil infiltration (Figure 1D,E). Of note, the expression of other cytokines like IL‐10 and IL‐22 was also diminished in *Ahr*‐deleted mice (Figure 1F,G). Finally, histological examination of the joints in control (upon PBS injection) mice and following serum transfer in wild type and *Ahr* KO animals confirmed that mutant mice exhibited less inflammation and bone erosion. Thus, these data suggest that AHR contributes to the development of serum‐induced arthritis. As IL‐22 secretion was also reduced in *Ahr* KO mice, we investigated the potential protective function of this cytokine.

### 3.2 Anti‐inflammatory properties of IL‐22 in serum‐induced arthritis

**FIGURE 2:**
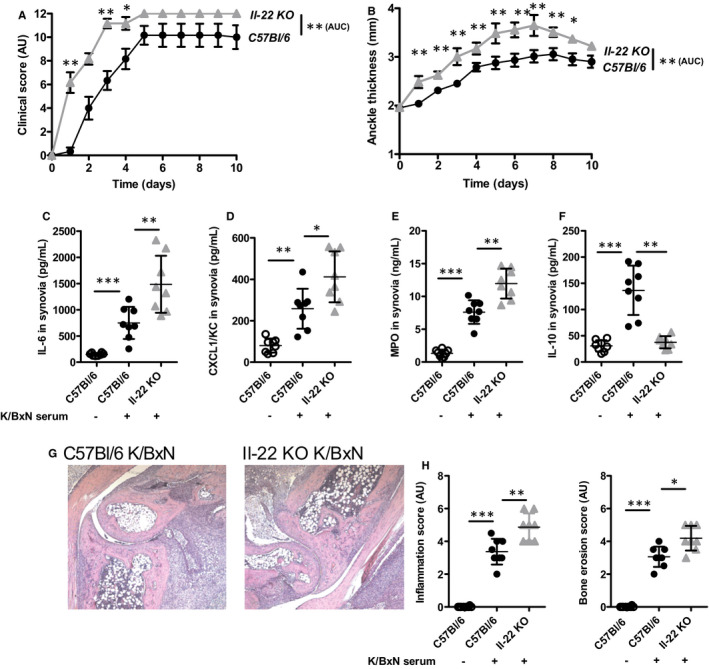
Exacerbated K/BxN serum arthritis in the absence of *Il‐22*. A, Clinical score (expressed in arbitrary units, AU) and B, ankle swelling (in mm). Cytokine expression in the synovium C, IL‐6 (in pg/mL), D, CXCL1 (in pg/mL), E, MPO (in ng/mL) and F, IL‐10 (in pg/mL) at day 6. G, Representative histological pictures of the joint following K/BxN serum transfer in wild type (C57Bl/6) and *Il‐22* KO animals. H, Inflammation and bone erosion scores (in arbitrary units, AU) quantified from histological analysis at day 10. Mean values +/− standard deviation are shown (n = 8 to 10 mice/group). Representative data from two independent experiments are shown. Data were analysed following a Mann‐Whitney *U* test. *P <.05**P <.01***P <.001. AUC: area under the curve

AHR‐dependent reduction of synovial IL‐22 was in line with the description of *Il‐22* as an AHR target gene.^5^, ^34^ However, the role of IL‐22 in the pathophysiology of arthritis remains debated, with RA patients showing increased IL‐22 levels in the plasma and the synovial fluid and anti‐IL‐22 antibodies injections in animals being either protective or detrimental, depending on the injection time, before or after the onset of the disease.^34^ This prompted us to investigate the impact of *Il‐22* gene deletion in the serum transfer model. In this setting, we observed worsened arthritis manifestations, with increased clinical score and paw swelling (Figure 2A,B) in K/BxN serum‐triggered *Il‐22*‐deficient mice. These observations were accompanied by increased IL‐6, CXCL1 and MPO detection in the synovium of *Il‐22* KO animals (Figure 2C‐E) and low IL‐10 production (Figure 2F). Accordingly, joint inflammation and bone damage were more severe in *Il‐22*‐deleted mice (Figure 2G,H), suggesting anti‐inflammatory effects of IL‐22. These data appear to contradict previous reports showing reduced severity of collagen‐induced arthritis and synovitis upon antigen‐induced arthritis in *Il‐22* KO mice,^35^, ^36^ which are more in favour of inflammatory functions for IL‐22. Furthermore, we confirmed these data using injections of an anti‐IL‐22 blocking antibody which also enhanced the severity of K/BxN serum arthritis (Figure S1A‐H).

### 3.3 *Ahr* and *Il‐22* expression in synovial tissue upon K/BxN serum challenge

**FIGURE 3:**
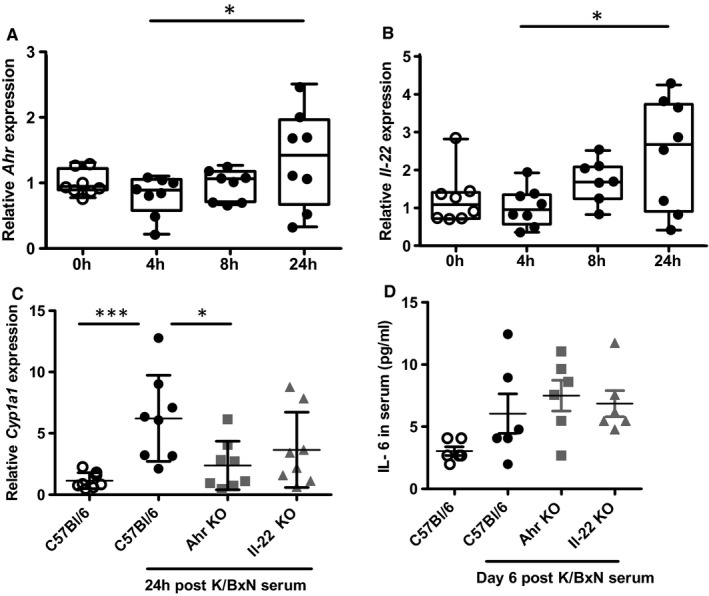
K/BxN serum transfer triggers *Ahr*‐dependent gene expression. A, *Ahr* and B, *Il‐22* expression in the synovium upon serum transfer up to 24h. C, *Ahr‐*dependent, *Il‐22*‐independent expression of *Cyp1a1* in the synovium following serum transfer at 24h and D, IL‐6 expression in the serum following K/BxN serum transfer in control (C57Bl/6), *Ahr* KO and *Il‐22* KO mice at day 6. Mean values ± standard deviation are shown (n = 6 to 8 mice/group). Data were analysed following a Mann‐Whitney *U* test. *P <.05***P <.001

Next, we sought to investigate the impact of arthritogenic K/BxN serum injection on AHR‐dependent signalling. We first noted an increased expression of the *Ahr* gene in the synovium 24h after serum injection before the appearance of clinical signs of arthritis (Figure 3A), which likely accounts for increased expression of known AHR‐dependent target genes expression such as *Il‐22* and *Cyp1a1* (Figure 3B,C). Finally, we noted that serum IL‐6 levels were unmodified in *Ahr*‐ and *Il‐22*‐deficient mice following arthritis induction (Figure 3D). This observation indicates that the AHR‐dependent control of the inflammatory response relies more on local, cell‐specific interactions within the joint microenvironment, rather than a systemic impairment of immune functions.

### 3.4 Promoting AHR signalling reduces inflammatory features upon K/BxN serum transfer

**FIGURE 4:**
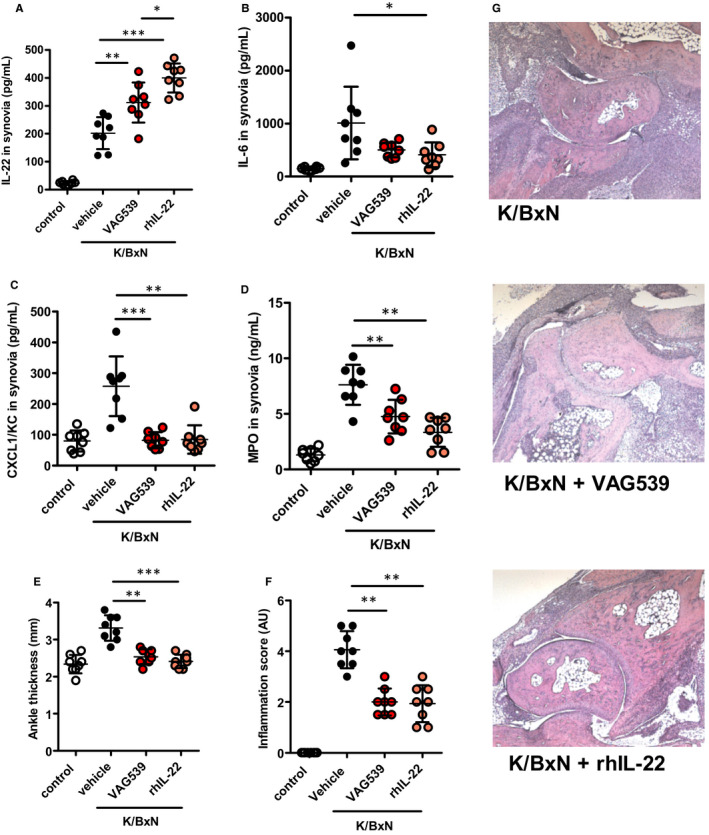
AHR agonist and rhIL‐22 attenuate arthritis severity following K/BxN serum transfer. A, Synovial IL‐22 (in pg/mL), B, IL‐6 (in pg/mL), C. CXCL1 (in pg/mL), D, MPO (in ng/mL) at day 6. E, Ankle thickness (in mm) and F, Inflammation score (in arbitrary units‐AU) were quantified in unchallenged controls (C57Bl/6) mice and following K/BxN serum transfer after vehicle injection or oral gavage with VAG539 or rhIL‐22 injection. G, Representative pictures of joint histology upon K/BxN serum transfer in controls (vehicle) or in VAG539‐ or rhIL‐22‐treated mice at day 10. Mean values +/− standard deviation are shown (n = 8 mice/group). Representative data from two independent experiments are shown. Data were analysed following a Mann‐Whitney *U* test. *P <.05**P <.01***P <.001

Protection against serum‐induced arthritis in *Ahr*‐deficient mice likely reflects a reduced expression of major pro‐inflammatory cytokines and chemokines (such as CXCL1) masking the loss of IL‐22 anti‐inflammatory properties in the animals. Therefore, we tested the impact of a pharmacological activation of AHR with a synthetic agonist, VAG539,^27^ to promote IL‐22 secretion and evaluate its protective effects on serum‐induced arthritis. In parallel, we injected recombinant human IL‐22 (rhIL‐22) to mice in which joint inflammation was also triggered by K/BxN serum injection. As expected, providing mice VAG539 orally stimulated *Il‐22* expression upon serum transfer, to such an extent that IL‐22 levels in the synovium almost reached those detected following rhIL‐22 injection (Figure 4A). Unexpectedly however, AHR activation failed to modify IL‐6 secretion in the synovia and exogenous supply of rhIL‐22 even led to a significant reduction of IL‐6 secretion. Nevertheless, low IL‐6 levels, associated to reduced CXCL1 (Figure 4C) and subsequent low neutrophil infiltration visualized through a marked decrease of MPO (Figure 4D) indicate that AHR activation and IL‐22 supplementation represent a powerful anti‐inflammatory approach. This is confirmed by reduced joint swelling (Figure 4E) and diminished joint inflammation revealed by histological analysis (Figure 4F,G) which reveal a marked improvement of arthritis following oral VAG539 or rIL‐22 injections prior to the injection of the arthritogenic serum. Of note, delayed (3 days after arthritis induction) administration of VAG539 still reduced clinical and biological symptoms in wild‐type mice (Figure S2). Finally, VAG539 injections in K/BxN‐treated *Il‐22* deficient mice did not improve arthritis symptoms (Figure S3), demonstrating that IL‐22 production is required downstream of AHR activation to promote protective effects.

### 3.5 Innate lymphoid cells are increased and contribute to acute joint inflammation

**FIGURE 5:**
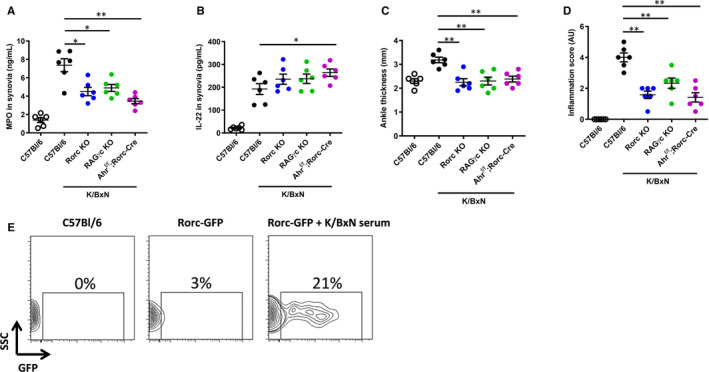
ILC3 are important players in acute arthritis. A, Synovial MPO (in ng/mL) and B, IL‐22 (in pg/mL), C, Ankle thickness (in mm) and D, Inflammation score (in arbitrary units, AU) were determined in control (following PBS injection) wild type (C57Bl/6) mice or upon K/BxN serum transfer in C57Bl/6, *Rag‐2* KO, *RORc* KO, *RORc* KO; *Rag‐2*, *Il‐2Rγc* mutants (RAGγc) and *Ahr* ^f/f^; Rorc‐*Cre* mice. E. representative picture of a FACS analysis showing GFP + cells harvested from the joints of control (C57Bl/6) non‐injected mice and Rorc‐GFP transgenic animals without or upon K/BxN serum injection at day 4. Mean values ± standard deviation are shown (n = 6 mice/group). Representative data from two independent experiments are shown. Data were analysed following a Mann‐Whitney *U* test. *P <.05**P <.01. SSCside scatter

Finally, we aimed at better defining the cellular mechanisms substantiating AHR‐ and IL‐22‐dependent protection against acute joint inflammation. For this, we first analysed molecular (MPO, IL‐22 secretion) and phenotypic (joint swelling, histological score) markers of inflammation in *Rorc* deficient mice, which lack a Th17 polarization response. Th17 cells also express IL‐22,^37^ and their differentiation depends on the RORγt transcription factor encoded by the *Rorc* gene.^38^ As seen in Figure 5A‐D, K/BxN‐injected *Rorc*‐deficient animals exhibit a markedly reduced inflammation compared to wild‐type controls, as demonstrated by lower MPO levels, joint swelling and inflammatory infiltrate and synovial Il‐22 levels were slightly elevated. K/BxN serum transfer arthritis is known to be independent of both B and T cells, external antigen and adjuvant for disease development.^39^ We confirmed that serum‐induced arthritis is independent of B and T cells using RAG‐deficient mice (Figure 5). Furthermore, inflammation induced by K/BxN serum transfer has been shown to remain unmodified in *Il‐17A*‐deleted mice,^40^ though other report challenged this observation.^41^ Importantly, both Th17 cells and ILC3 differentiation depend on the transcription factor RORγt. To discriminate their involvement on K/BxN‐triggered joint inflammation, we next used mice (dubbed here RAGc mice) that, in addition to a deletion of the *Rorc* gene, also harbour mutations in genes encoding Rag‐2 and the Il‐2 receptor γ chain, thus lacking B and T cells as well as all innate lymphoid cells (ILC). RAGc mice exhibited reduced arthritis and inflammation appeared undistinguishable from *Rorc* KO animals (Figure 5A‐D), claiming for a major effect emanating from pro‐inflammatory ILCs that are impaired in these mice. ILC3 cells are likely such player, being both high producers of IL‐22^42^ and dependent on AHR for their ontogeny.^16^

To ascertain that the inflammatory response was mediated by *Rorc* expressing cells, we used Rorc‐GFP reporter mice and found a rapid and massive (21%) recruitment of GFP^+^ ILC3 cells (gating strategy is shown in Figure S4) in the inflamed synovia on day 4 following K/BxN serum challenge (Figure 5E). To investigate the role of these cells in acute arthritis, we used knock‐out mice in which conditional deletion of the *Ahr* floxed (f/f) gene was induced by a Rorc‐driven Cre transgene (*Ahr^f/f^xRorc‐Cre*). Again, inflammation appeared less damageable in these animals, which are featured by reduced MPO levels, ankle swelling and inflammatory cells infiltration. Interestingly, synovial IL‐22 secretion was significantly augmented, suggesting AHR‐dependent negative regulation of the *Il‐22* gene in ILC3 cells, as opposed to Th17 cells. Such involvement of AHR and IL‐22 in ILC is consistent with our observations that both *Ahr* and *Il‐22* deletions are characterized by rapid (within 2 days) improvement (*Ahr* KO, Figure 1A‐B) or worsening (*Il‐22* KO, Figure 2A‐B) of joint inflammation following K/BxN serum injection.

Altogether, these data provide reasonable argument in favour of a positive, protective function of IL‐22 secretion and role of innate cells such as ILC3 cells in the acute phase of joint inflammation.

## 4 DISCUSSION

**FIGURE 6:**
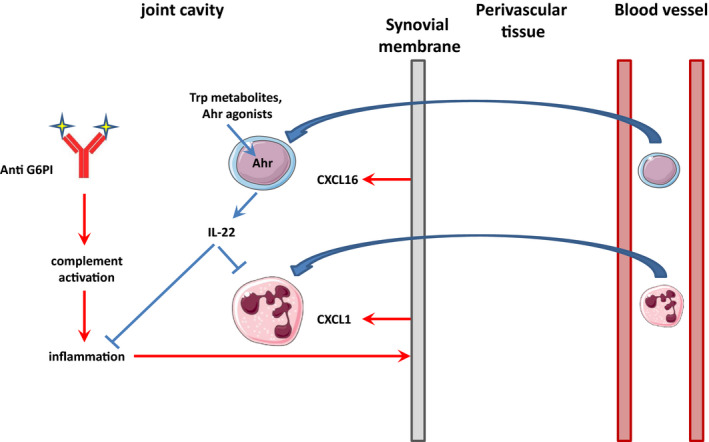
A model integrating ILC3 in serum‐dependent acute arthritis. The cartoon illustrates that complement‐dependent inflammation triggered by anti‐glucose‐6‐phosphate isomerase (G6PI) antibodies/G6PI immune complexes activate cells of the synovial membrane. In response, the cells secrete chemokines such as CXCL1 and CXCL16 attracting neutrophils and ILC3s. Once activated, ILC3 secrete IL‐22 which dampens inflammation and neutrophil recruitment

With 1% of the world population being affected, rheumatoid arthritis is the most frequent chronic inflammatory arthritis.^43^ In addition to its high prevalence, RA also illustrates the nature of ‘complex diseases’, associating genetic factors and environmental triggers.^44^ So far, variants in more than 100 genes along with all immune cells of the innate and adaptive immune systems have been studied and involved in RA pathogenesis.^45^ An important feature of RA is the initiation phase, driven by the infiltration of innate inflammatory cells and their secretion of cytokines such as TNF, IL‐6, IL‐1 and IL‐17, which feed and amplify the subsequent B‐ and T cell–dependent cartilage and bone erosion. Innate lymphoid cells (ILCs) are recently classified lymphocytes that include, among others, cytokines‐secreting cells that mirror the polarized CD4^+^ helper T‐cell subsets Th1, Th2 and Th17, called ILC1, ILC2, and ILC3, respectively and dubbed “helper‐like ILCs”.^46^ Previous reports have considered the role of NK cells (which are now classified as ILCs) in the pathogenesis of RA through their interactions with monocytes.^47^ Interestingly, the NK cell marker CD56 is also shared by ILC1 cells, which are enriched in the synovial fluid of RA patients.^48^ Similar observations were made for NKp44^+^ ILC3‐like cells whose enrichment is positively correlated with disease activity. Importantly, these ILC3‐like cells were a source of IL‐22 and TNFα.^49^ However, whether these cells accumulation is the cause or the consequence of the local inflammation is still unknown. With regards to IL‐22 which is mainly produced by ILC3 (but also by CD4 + T cells^50^ and T‐cells^51^), its role in RA aetiology is similarly debated, as various experimental setting have demonstrated either protective or pathogenic effects.^52^ Such duality of a cytokine's function has been reconciled by considering that the same role (ie proliferation, inhibition of apoptosis) can provide protection in an acute setting for instance by promoting regulatory cells, whereas FLS proliferation leading to synovial hyperplasia drives chronic RA pathogenesis. To explore in more details the role of ILC3 and IL‐22 in acute joint inflammation, we analysed the arthritogenic effects of K/BxN serum transfer in *Ahr* (a major determinant of ILC3 ontogeny^53^)‐and *Il‐22*‐deficient animals. Our results show that serum‐induced cytokine expression is significantly reduced in *Ahr* KO mice, in line with diminished macroscopic and histological manifestations of inflammation. Conversely, *Il‐22* KO mice (as well as mice in which an anti‐IL‐22 neutralizing monoclonal antibody was injected) exhibited worsened symptoms (Figures 1, 2 and S1). Intriguingly, similar effects (protection against arthritis) could be noticed in *Ahr* KO animals and upon AHR activation with the VAG539 agonist (Figures 1 and 4). We observed that gene ablation affects the expression of most pro‐inflammatory cytokines and chemokines, as well as anti‐inflammatory mediators (IL‐10, IL‐22). In this case, protection is likely caused by the reduction of a pro‐inflammatory environment. In contrast, VAG539 appears to specifically increase IL‐22 production and drive anti‐inflammatory conditions. It is possible that engagement of this agonist with AHR induces conformational changes of the receptor leading to the activation of pathways particularly controlling Il‐22 expression, while *Ahr* ablation eliminates NF‐κB signalling, which is upstream of most immune genes. Of note, in both cases, the impact of the gene deletion could be visible shortly (24h) after serum transfer, indicating that innate cells were more likely to account for the phenotype, rather than T‐ or B cells which require several days for their differentiation and activation. These data point to a major anti‐inflammatory effect of IL‐22, which was further confirmed by injections of the recombinant cytokine (rhIL‐22, Figure 4). Two major cell types are known to produce this cytokine: Th17 cells and ILC3. As mentioned above, the rapid effects seen in *Il‐22* KO animals (Figure 2) and following rhIL‐22 administration (Figure 4), as well as the fast expansion of *Rorc*‐GFP + cells upon serum transfer (Figure 5E) suggest that ILC3 play a major role in this process. This hypothesis is strongly supported by our observations that *Rorc* KO mice (which lack both Th17 and ILC3), Rag/Il‐2R γ chain mutants combined to *Rorc* deletion (lacking all T cells and ILC3) exhibited a similar phenotypes (with regards to molecular and histological signs of inflammation) compared to mice devoid of ILC3 only, due to a targeted deletion of a floxed *Rorc* gene in *Ahr*‐expressing cells (Figure 5). Importantly, wild type (C57Bl/6) and *Rag‐2* KO (lacking B and T cells) exhibited similar phenotypes. A cartoon summarizing these events is shown in Figure 6. We propose a hypothetical mechanism in which complement‐dependent inflammation triggered by immune complexes present in K/BxN serum is down modulated by IL‐22‐producing ILC3. However, our data do not rule out a possible involvement of other innate cells, such as neutrophils which are also capable of IL‐22 secretion,^54^ and accounting for the unaltered IL‐22 expression level in the synovial fluid of T cells‐ and ILC3‐depleted animals (Figure 5B). Future experiments aiming at depleting ILC3 with an *Ahr*‐driven diphteria toxin transgene might be informative in this regards. We also demonstrate in this work that AHR signalling and subsequent *Il‐22* expression is induced upon K/BxN serum injection (Figure 3). However, the molecular details of AHR activation are not identified at this stage and more work will be needed to identify the endogenous ligands responsible for the induction of AHR signalling. Serum transfer arthritis is modified in *Indoleamine 2,3‐dioxygenase 2* (*Ido2*) mutant mice,^55^ suggesting that still to be identified endogenous tryptophan metabolites might account for this observation.

Altogether, our data indicate that AHR is an interesting hub capable of sensing exogenous (pollutants or microbial derivatives), as well as endogenous molecules, providing an important surveillance system to sample environmental changes that may trigger acute inflammatory responses. Hence, pharmacological targeting (with agonists, as shown in this report, Figure 4) of this receptor might offer novel opportunities to reduce acute inflammation.

## CONFLICT OF INTEREST

D. Togbe and L. Fauconnier are employees at ArtImmune. The authors have declared that no conflict of interest exists.

## AUTHOR CONTRIBUTION

**Ramzi Nehmar:** Conceptualization (equal); Formal analysis (equal); Investigation (equal). **Louis Fauconnier:** Investigation (equal). **josé carlos alves filho:** Investigation (equal). **dieudonnée togbe:** Investigation (equal). **Aurore DeCauwer:** Investigation (supporting); Methodology (supporting). **siamak bahram:** Funding acquisition (equal); Writing‐original draft (equal). **Marc Le Bert:** Investigation (supporting). **Bernard Ryffel:** Conceptualization (equal); Data curation (equal); Formal analysis (equal); Funding acquisition (equal); Project administration (equal); Supervision (equal); Writing‐original draft (equal); Writing‐review & editing (equal). **Philippe Georgel:** Conceptualization (equal); Data curation (equal); Formal analysis (equal); Project administration (equal); Supervision (equal); Validation (equal); Writing‐original draft (equal); Writing‐review & editing (equal).

## Supporting information

Fig S1‐S4

## Data Availability

The data that support the findings of this study are available from the corresponding author upon reasonable request.
