# Supplementary material for: Aryl hydrocarbon receptor (Ahr)‐dependent Il‐22 expression by type 3 innate lymphoid cells control of acute joint inflammation
Source: J Cell Mol Med. 2021 Mar 18;25(10):4721–31. doi: 10.1111/jcmm.16433 (PMC8107095; doi:10.1111/jcmm.16433)

**Supplemental Figure 1. Exacerbated K/BxN serum transfer arthritis upon anti-IL-22 antibody administration**. **A.** Clinical score (expressed in arbitrary units, AU) and **B.** ankle swelling (in mm). Cytokine expression in the synovium **C.** IL-6 (in pg/ml), **D.** CXCL1 (in pg/ml), **E.** MPO (in ng/ml) and **F.** IL-10 (in pg/ml) at day 6. **G.** Representative histological pictures of the joint following K/BxN serum transfer in wildtype (C57Bl/6) with or without anti-IL-22 antibody (mAb) administration. **H.** Inflammation and bone erosion scores (in arbitrary units, AU) quantified from histological analysis at day 10. Mean values +/- standard deviation are shown (n=6 mice/group). Representative data from two independent experiments are shown. Data were analyzed following a Mann-Whitney U test. * p<0.05. ** p<0.01. ***p<0.001.


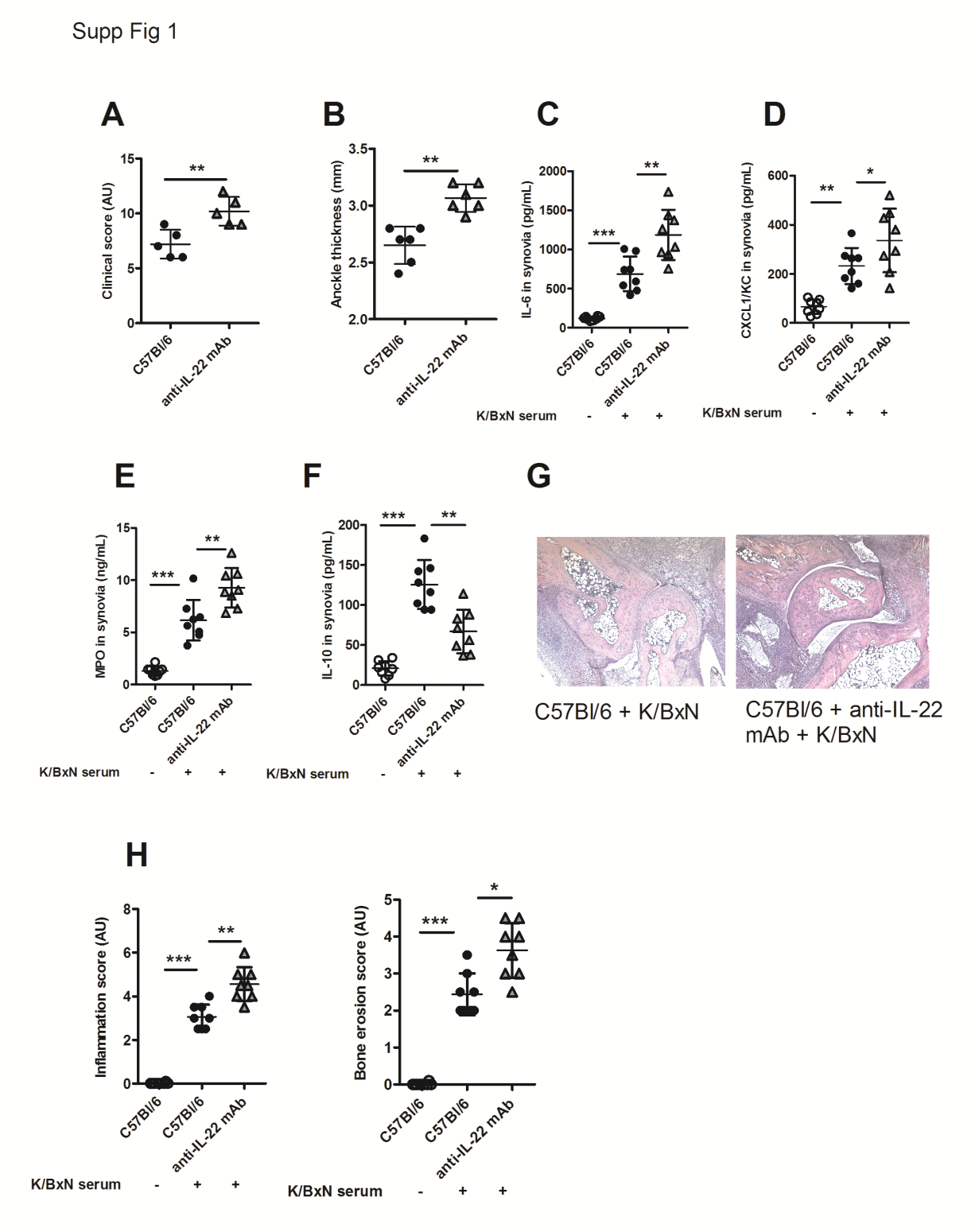


**Supplemental Figure 2. Delayed administration of the AHR agonist VAG539 attenuates arthritis severity following K/BxN serum transfer. A.** Synovial IL-22 (in pg/ml), **B.** IL-6 (in pg/ml), **C.** CXCL1 (in pg/ml), **D.** MPO (in ng/ml) at day 6. **E.** Ankle thickness (in mm) and **F.** Inflammation score (in arbitrary units-AU) were quantified in unchallenged controls (C57Bl/6) mice and following K/BxN serum transfer after vehicle or oral gavage with VAG539 at day 3. **G.** Representative pictures of joint histology upon K/BxN serum transfer in vehicle- or in VAG539-treated mice at day 10. Mean values +/- standard deviation are shown (n=8 mice/group). Representative data from two independent experiments are shown. Data were analyzed following a Mann-Whitney U test. * p<0.05.


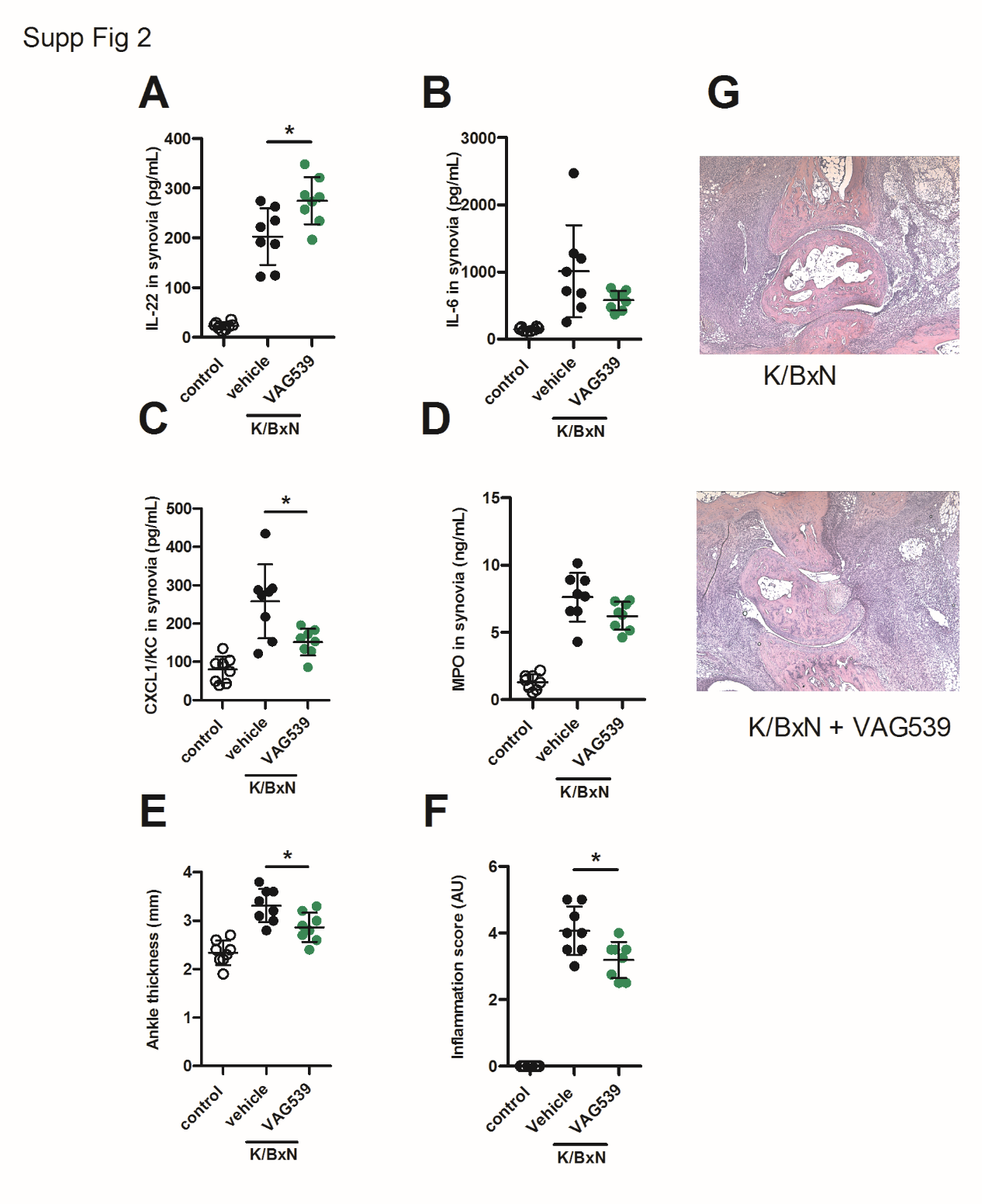


**Supplemental Figure 3. AHR agonist VAG539 activity is IL22-dependent in the K/BxN serum transfer arthritis.** **A.** Synovial IL-22 (in pg/ml), **B.** IL-6 (in pg/ml), **C.** CXCL1 (in pg/ml), **D.** MPO (in ng/ml) at day 6. **E.** Ankle thickness (in mm) and **F.** Inflammation score (in arbitrary units-AU) were quantified in control (no serum) and following K/BxN serum transfer of untreated mice or after oral gavage with VAG539 or vehicle (PBS) in *Il-22*-deficient mice. **G.** Representative pictures of joint histology upon K/BxN serum transfer in non treated, VAG539-treated and PBS-treated *Il-22* KO mice at day 10. Mean values +/- standard deviation are shown (n=6 mice/group). Representative data from two independent experiments are shown. Data were analyzed following a Mann-Whitney U test and no significant difference between groups of K/BxN-injected arthritic mice was noted.


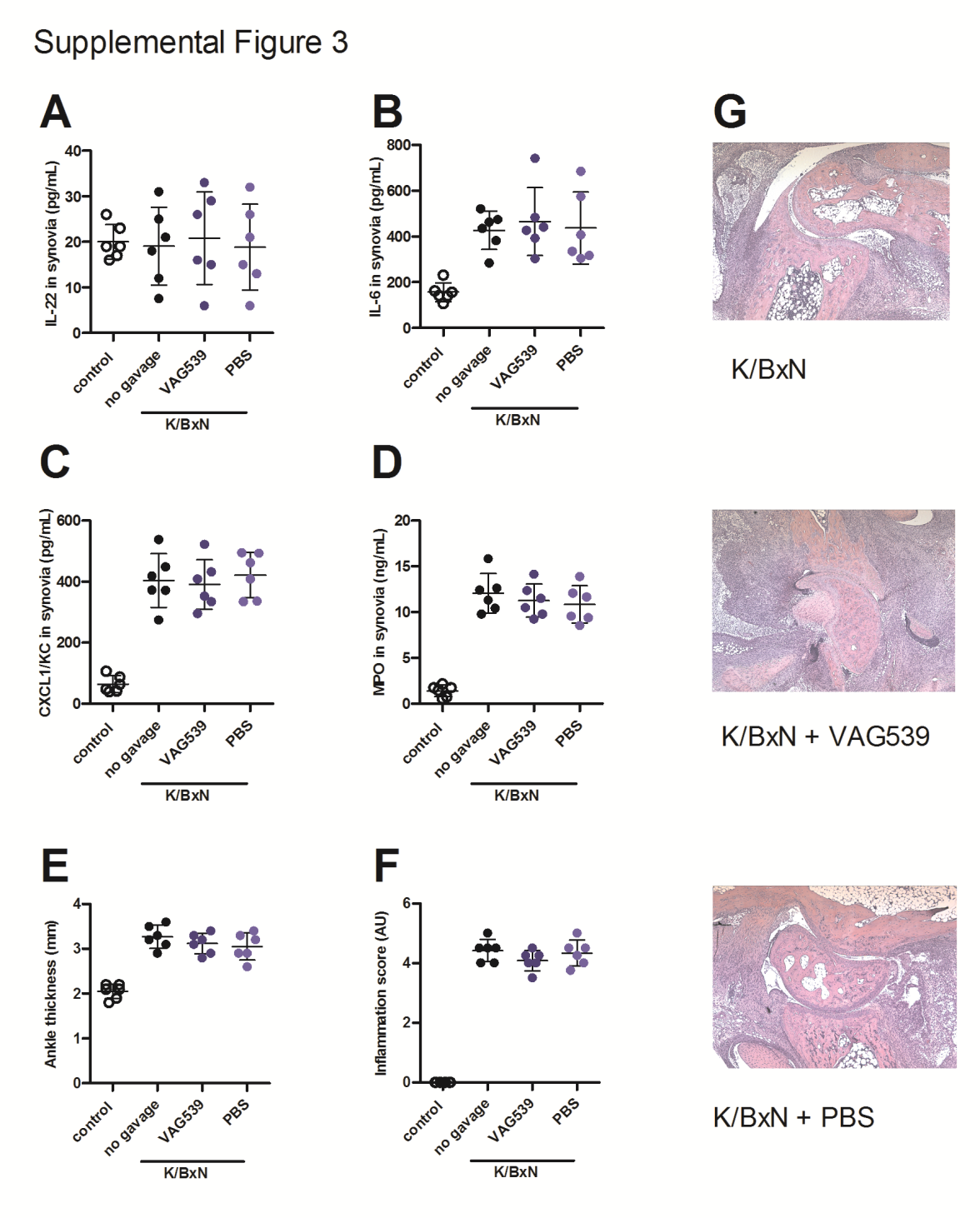


**Supplemental Figure 4. Gating strategy to identify GFP+ ILC3 cells in the synovium.** Synovial cells were isolated from GFP-Rorγt reporter mice and enriched for ILC3 using the sequential elimination of lineage markers to obtain ILC3 expressing GFP.


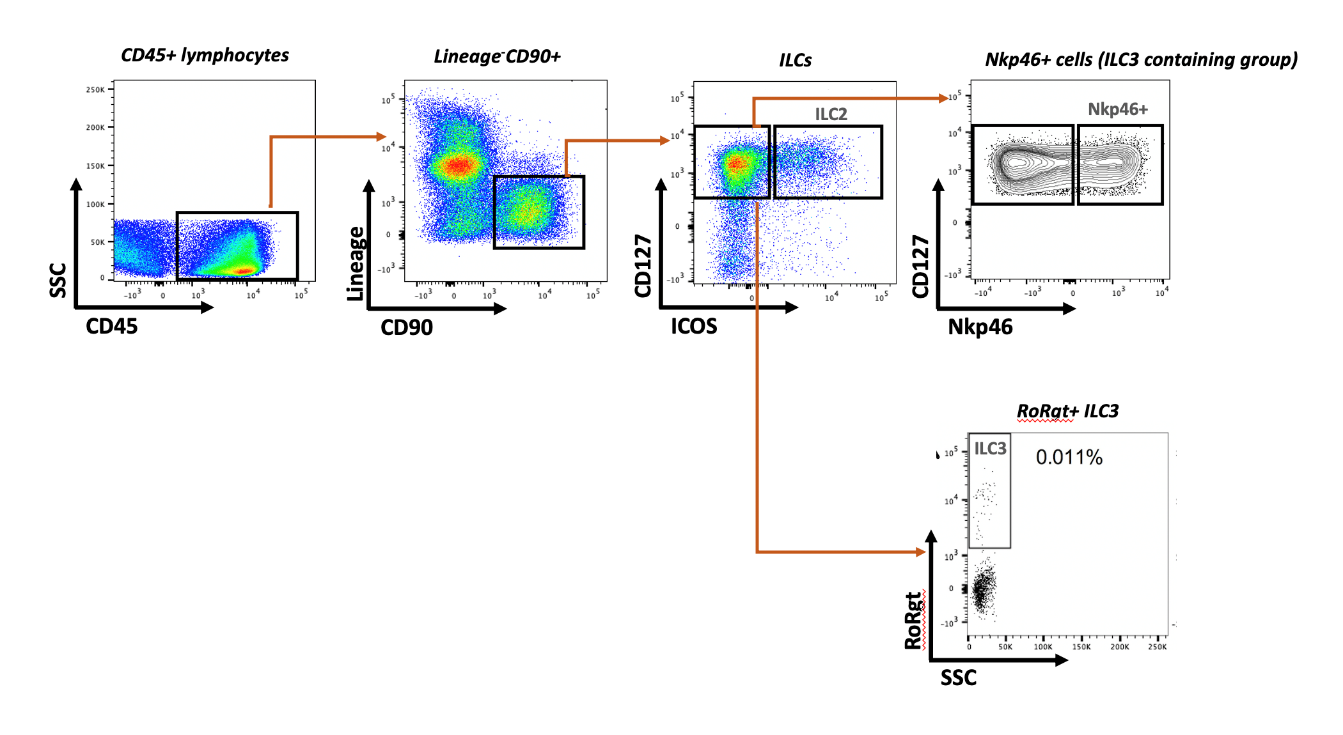

Supplement: Supplementary file 1 — Fig S1‐S4 [file JCMM-25-4721-s001.docx]
